# Pterostilbene Attenuates Particulate Matter-Induced Oxidative Stress, Inflammation and Aging in Keratinocytes

**DOI:** 10.3390/antiox10101552

**Published:** 2021-09-29

**Authors:** Wei-Lin Teng, Pao-Hsien Huang, Hui-Chun Wang, Chih-Hua Tseng, Feng-Lin Yen

**Affiliations:** 1 Graduate Institute of Natural Products, College of Pharmacy, Kaohsiung Medical University, Kaohsiung 807, Taiwan; u109831001@kmu.edu.tw; 2 Department of Fragrance and Cosmetic Science, College of Pharmacy, Kaohsiung Medical University, Kaohsiung 807, Taiwan; R040035@gap.kmu.edu.tw (P.-H.H.); chihhua@kmu.edu.tw (C.-H.T.); 3 Drug Development and Value Creation Research Center, Kaohsiung Medical University, Kaohsiung 807, Taiwan; 4 Department of Medical Research, Kaohsiung Medical University Hospital, Kaohsiung 807, Taiwan; 5 Department of Marine Biotechnology and Resources, National Sun Yat-Sen University, Kaohsiung 807, Taiwan; 6 School of Pharmacy, College of Pharmacy, Kaohsiung Medical University, Kaohsiung 807, Taiwan; 7 Department of Pharmacy, Kaohsiung Municipal Ta-Tung Hospital, Kaohsiung 807, Taiwan; 8 Institute of Biomedical Sciences, National Sun Yat-Sen University, Kaohsiung 807, Taiwan

**Keywords:** particulate matter, pterostilbene, reactive oxygen species, keratinocyte, inflammation, aging, skin penetration

## Abstract

Particulate matter (PM) is the main indicator of air pollutants, and it may increase the level of reactive oxygen species (ROS) in keratinocytes, leading to skin inflammation, aging, and decreased moisturizing ability. Pterostilbene (PTS) is a dimethylated analog of resveratrol that has antioxidant effects. However, the molecular mechanisms of PTS in preventing PM-induced keratinocyte inflammation and aging have not been investigated yet. Therefore, we used PM-induced human keratinocytes to investigate the protective mechanisms of PTS. The results showed that 20 μM PTS had no toxicity to HaCaT keratinocytes and significantly reduced PM-induced intracellular ROS production. In addition, nuclear translocation of the aryl hydrocarbon receptor (AHR) was inhibited by PTS, leading to reduced expression of its downstream CYP1A1. PTS further inhibited PM-induced MAPKs, inflammation (COX-2), and aging (MMP-9) protein cascades, and rescued moisturizing (AQP-3) protein expression. We analyzed the PTS content in cells at different time points and compared the concentration required for PTS to inhibit the target proteins. Finally, we used the skin penetration assay to show that the PTS essence mainly exists in the epidermal layer and did not enter the system circulation. In conclusion, PTS could protect HaCaT keratinocytes from PM-induced damage and has the potential to become a cosmetic ingredient.

## 1. Introduction

According to the World Health Organization (WHO), ambient air pollution has become a major health problem that causes 4.2 million deaths every year. Particulate matter (PM) is considered to be one of the main air pollutants by the National Ambient Air Quality Standard (NAAQS) [1]. PM is composed of organic mass, elemental mass, atmospheric semi-volatile species, crustal materials and some biological materials [2]. In the past decades, many epidemiological and toxicological studies have confirmed that PM affects the main organs of the body, such as the lungs, immune system, cardiovascular system, nervous system and integumentary system [2,3,4,5]. Human skin is the first defense against air pollutants, and PM has been demonstrated to induce oxidative stress by diminishing skin barrier function and induce skin inflammation; therefore, PM overexposure has been positively correlated with skin aging, psoriasis, and atopic eczema [6].

Aryl hydrocarbon receptor (AHR) is an important chemical sensor and a latent transcription factor which is highly expressed in all skin cells [7]. AHR can be activated in the skin by organic components in PM, such as polycyclic aromatic hydrocarbons (PAHs) [8]. AHR activation increases the production of reactive oxygen species (ROS) and expression of matrix metalloproteinases (MMPs), leading to the degradation of collagen and elastin, which cause skin aging [8,9,10,11,12]. In addition, PM has been identified to induce the expression of COX2, leading to cell inflammation through intracellular ROS production and activation of mitogen-activated protein kinases (MAPKs; including phospho-p38, phospho-JNK and phospho-ERK) and NK-κB in keratinocytes [6,13,14,15]. Many publications have shown that after PM overexposure, various compounds from natural products in a topical antioxidant formulation could effectively inhibit AHR translocation and inflammation, leading to the repair of skin barrier function via an antioxidant effect [16,17,18].

Pterostilbene (PTS) is a natural stilbenoid and a dimethylated analog of resveratrol, which is mainly found in *Pterocarpus marsupium* [19]. PTS has anti-inflammatory, antioxidant, anti-tumor, neuroprotective and anti-diabetic activities [19,20]. In the skin, PTS can scavenge UVB-induced ROS and protect against photo-damage through the activation of the Nrf2/ARE pathway [21]. In addition, PTS can reduce allergic contact dermatitis by preventing cell apoptosis and inhibiting the activation of inflammasomes [22]. However, the molecular biological mechanisms of PTS in preventing oxidative stress, inflammation and aging of keratinocytes exposed to PM has not yet been published.

Our current study aimed to observe PM-induced keratinocyte inflammation and inhibit aging by PTS treatment. We also used ex vivo skin penetration to determine the skin absorption of PTS in a topical antioxidant formulation. We further evaluated the intracellular concentration of PTS required to effectively prevent PM-induced skin damage through cellular uptake experiments. We demonstrated that PTS treatment can be used to protect keratinocytes against PM-induced inflammation and aging. In addition, we further evaluated the intracellular concentration of PTS to effectively prevent PM-induced skin damage through cellular uptake experiments.

## 2. Materials and Methods

### 2.1. Cell Culture and Viability Assay

The HaCaT cell line of human keratinocytes was obtained from the Istituto Zooprofilattico Sperimentale della Lombardia e dell’Emilia Romagna (Brescia, Italy). HaCaT cells were maintained in DMEM (Himedia Laboratories, Mumbai, India) containing 10% fetal bovine serum (Hazelton Product, Denver, PA, USA) and 1% penicillin–streptomycin–amphotericin B solution (PSA; Biological Industries, Cromwell, CT, USA) and human skin fibroblast cell line (CCD-966SK) was obtained from the Food Industry Research and Development Institute (Hsinchu, Taiwan). CCD-966SK cells were maintained in MEM (Thermo Fisher Scientific, Waltham, MA, USA) with 0.1 mM non-essential amino acids (NEAA; Thermo Fisher Scientific) containing 10% fetal bovine serum. Both cells were incubated at 37 °C in 5% CO_2_. 1 × 10^4^ HaCaT cells were suspended in 100 μL culture medium and seeded into 96-well plates for 24 h. Pterostilbene was purchased from Professor Chih-Hua Tseng and the purity of PTS (>95%) was determined by high performance liquid chromatography (HPLC) analysis. Different concentrations (5 to 80 μM) of PTS were prepared in DMEM without FBS, then added to each well for 24 h. After treatment, the medium was removed and 100 μL of 0.5% MTT solution was added to each well and incubated for 3 h under normal culture condition. The crystals were dissolved in DMSO and the absorbance of each well at 550 nm was evaluated by using a microplate spectrophotometer (Molecular Devices, San Jose, CA, USA).

### 2.2. Intracellular Reactive Oxygen Species (ROS) Assay

For detection of intracellular ROS, 1.5 × 10^4^ HaCaT cells were cultured in 96-well plates for 24 h under 37 °C and 5% CO_2_ condition. Cells were treated with 10 and 20 μM PTS for 6 h, and then reacted with 20 μM dichlorodihydrofluorescein diacetate (DCFH-DA; Sigma, Tokyo, Japan) solution for 30 min. Next, 50 μg/cm^2^ PM (Standard Reference Material^®^ 1649b; Gaithersburg, MD, USA) was added and incubated for 1 h. After that, cells were washed twice with PBS and fluorescence intensity was detected by a fluorescent plate reader (BioTek, Winooski, VT, USA), with excitation and emission wavelengths of 485 and 528 nm, respectively.

### 2.3. Western Blot

This was performed in accordance with a previous study of PM-induced HaCaT cells model [23]. In a 6 cm dish, 1 × 10^6^ cells were cultured for 24 h. PM (50 μg/cm^2^) was prepared in PBS and sonicated for 10 min. HaCaT cells were cultured in FBS free medium and treated with PTS before adding PM. After various time points, cells were lysed with RIPA Lysis Buffer (Merck Millipore, Burlington, MA, USA), then centrifuged at 12,000 rpm for 10 min. BCA protein assay kit (Thermo Fisher Scientific, Waltham, MA, USA) was used to determine protein concentration. Samples were separated by SDS-PAGE and transferred to a PVDF membrane (Merck Millipore). Membranes were blocked for 1 h and washed with Tris-buffered saline (TBS) with 1% Tween-20. Membranes were then incubated with primary antibodies including AhR (1:1000; #83200, Cell Signaling Technology, Danvers, MA, USA), COX-2 (1:1000; #12282, Cell Signaling Technology, Danvers, MA, USA), MMP-1 (1:1000; 10371-2-AP, Proteintech Group, Inc., Wuhan, China), MMP-2 (1:1000; #87809, Cell Signaling Technology, Danvers, MA, USA), MMP-9 (1:1000; ARG54980, Arigo Biolaboratories Corp., Hsinchu, Taiwan), Aquaporin 3 (1:1000; ARG10648, Arigo Biolaboratories Corp.), phospho-p38 (1:1000; #09-272, Merck Millipore), phospho-ERK (1:1000; #05-797R, Merck Millipore), phospho-JNK (1:1000; #4668, Cell Signaling Technology, Danvers, MA, USA), CYP1A1 (1:1000; A2159, ABclonal Techonlogy, Inc., Woburn, MA, USA), GAPDH (1:1000; sc47724, Santa Cruz Biotechnology, Dallas, TX, USA.), and Lamin B1 (1:1000; ARG65740, Arigo Biolaboratories Corp.) at 4 °C overnight. Next, membranes were incubated with HRP-conjugated secondary antibody for 1 h at room temperature and reacted with enhanced chemiluminescence reagents (ECL; Thermo Fisher Scientific). Protein signals were detected by Touch Imager (e-BLOT; Shanghai, China) and expression quantified by image J software.

### 2.4. HaCaT Cells Nuclear Protein Extraction

The experimental procedures for cell seeding, PM preparation and protein expression analysis were the same as the previous Western blot paragraph. HaCaT cells were incubated with PM (50 μg/cm^2^) for 0.5, 1, 2, 4 and 6 h. The nuclear proteins at each time point were collected by the Nuclear Extraction Kit (Signosis, Inc.; Santa Clara, CA, USA).

### 2.5. Measurement of Pterostilbene Content in HaCaT Cells

HaCaT cells were seeded in a 12-well plate for 24 h and then cultured with 20 μM PTS for various time points in FBS free medium. After treatment, the extracellular compartment (culture medium) was mixed with DMSO to dissolve PTS. For the intracellular compartment, cells were lysed by 0.5% SDS and washed with methanol, and then collected in a tube and sonicated for 30 min. After passing through a 0.45 um filter, the content of PTS in all samples were determined by high performance liquid chromatography (HPLC) Chromaster system with 5410 ultraviolet (UV) detector, 5210 Auto Sampler and 5110 Pump (Hitachi, Tokyo, Japan). The HPLC column was Mightysil RP-18 GP (250 × 4.6 mm, 5 μm; Kanto Chemical Co., Inc., Tokyo, Japan) and the mobile phase consisted of acetonitrile and double distilled water (35:65, *v*/*v*). The PTS detection wavelength of UV detector was 307 nm, and the retention time was approximately 4.3 min. The PTS content of all samples was calculated by comparing it with the PTS standard curve.

### 2.6. In Vitro Skin Penetration Study

In vitro skin penetration assay was performed according to the previous study and was modified from the guideline of the European Cosmetic and Perfumery Association (COLIPA) [24]. The skin of pig flank region purchased from a local market was used as the in vitro skin penetration experimental model. Skin samples without wounds, ulcers and abscesses were selected, and cut to an appropriate size about 2 × 2 cm^2^. Next, skin samples were placed on 10 mm Franz Diffusion Cells and stirred for 1000 rpm in Receptor Fluid at 32 °C. The test essence containing PTS (200 μL) were added to the donor chambers for 1, 2 and 4 h. After treatment, skin samples were taped-stripped 15 times with 3M adhesive tape to remove the stratum corneum. Next, skin samples were placed on a heater at 90 °C, and then the epidermis and dermis were separated using a scalpel. All samples were placed into methanol and sonicated for one hour to extract PTS. Finally, the content of PTS in stratum corneum, epidermis, dermis and non-penetration compartment were analyzed by HPLC. The test essence is composed of 0.0625% PTS, 0.13% Satiaxane™ VPC 930 (HonorChem Co, Ltd., Tainan, Taiwan), 6.25% DMSO, 25% Butylene Glycol (BG; WWRC Taiwan Co, Ltd., Kaohsiung, Taiwan) and 68.56% water.

### 2.7. Statistical Analysis

Experimental data were calculated and analyzed using Microsoft Excel 2016 software (Microsoft Office; Microsoft Corporation, Redmond, WA, USA) and SPSS software version 19 (SPSS Inc., Chicago, IL, USA). We used one-way ANOVA to compare data from multiple group and significant differences were determined by Tukey’s test. Data were expressed as mean ± SD and a *p*-value less than 0.05 was considered as a significant difference.

## 3. Results

### 3.1. Effects of Pterostilbene on Cell Viability and Intracellular ROS Generation in Human Keratinocyte Cells

**Figure 1:**
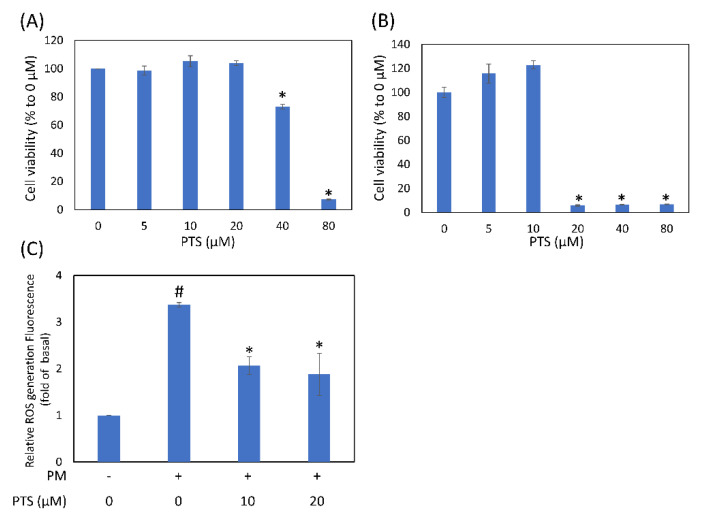
Effects of different PTS concentrations on human keratinocyte (**A**) HaCaT cell and (**B**) CCD-966SK dermal fibroblast cell viability and PM-induced intracellular ROS generation. (**A**) and (**B**) cell viability was determined by the MTT assay after 24 h of PTS treatment. (**C**) HaCaT cells were pre-treated with 10 and 20 μM PTS for 6 h and incubated with PM for 1 h. # *p* < 0.05 versus untreated control. * *p* < 0.05 versus PM treatment control.

Before using PTS on human skin as a cosmetic product, it is very important to confirm the toxicity of PTS in HaCaT cells. As shown in Figure 1A, 5 to 20 μM PTS did not affect HaCaT cell viability, while 40 μM PTS significantly inhibited cell viability by 27.0% and 80 μM PTS inhibited cell viability by 92.8%. In Figure 1B, 5 and 10 μM PTS did not affect CCD-966SK cell viability, but 20 μM PTS significantly inhibited cell viability. Figure 1C showed that intracellular ROS production induced by PM was significantly inhibited by 10 and 20 μM PTS. These results indicate that PTS concentration below 20 μM was not toxic to HaCaT keratinocytes and inhibited ROS production.

### 3.2. PTS Inhibited the Translocation of AHR into Nucleus

**Figure 2:**
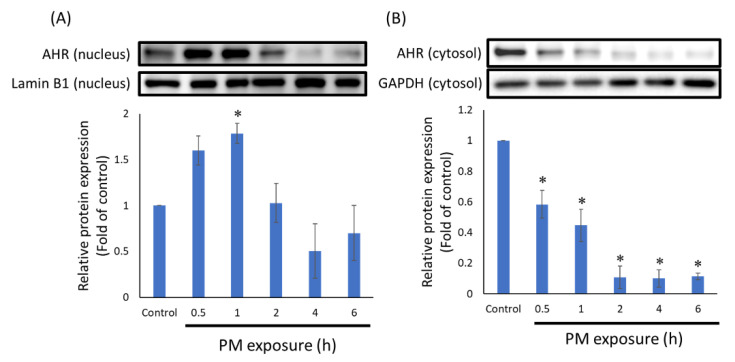
The time point of AHR protein translocation in keratinocytes exposed to PM. (**A**) AHR in the nucleus. (**B**) AHR in the cytosol. *n* = 3 for each group; * *p* < 0.05 compared with control.

**Figure 3:**
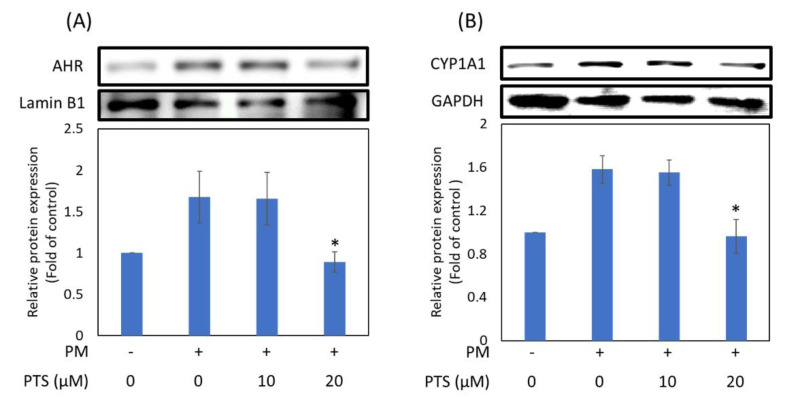
PTS inhibited AHR translocation and CYP1A1 expression. (**A**) AHR in nucleus and (**B**) CYP1A1 total protein. *n* = 3 for each group; * *p* < 0.05 compared with only PM exposure.

To understand whether PTS can inhibit translocation of AHR protein, we isolated the nuclear proteins from HaCaT cells and analyzed the expression level. In HaCaT cells exposed to PM, the AHR protein translocated into the nucleus at 0.5 h, and there was a significant difference in comparison with the control group at 1 h, as shown in Figure 2A. In addition, as the time of exposure to PM increased, the amount of AHR in the cytoplasm was also significantly reduced (Figure 2B). Therefore, in the next experiment, we induced HaCaT cells with PM for 0.5 h before treatment with PTS. As shown in Figure 3A, 20 μM PTS significantly inhibited nuclear AHR protein expression. In addition, the expression of CYP1A1 protein regulated by AHR was also significantly inhibited by 20 μM PTS (Figure 3B). These results indicate that PM-induced AHR nuclear translocation in HaCaT cells can be inhibited by PTS.

### 3.3. PTS Inhibited PM-Induced MAPKs Protein Activation

**Figure 4:**
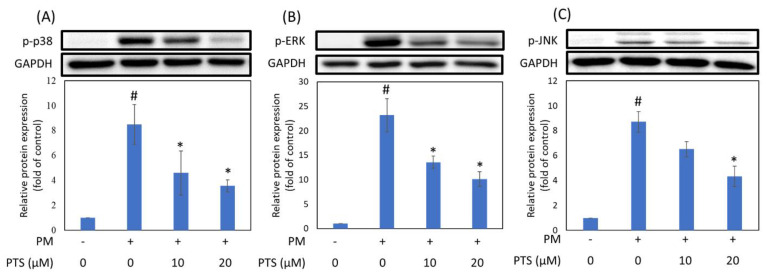
Expression of MAPKs in HaCaT keratinocytes exposed to PM and treated with PTS. Samples were pre-treated with PTS for 3 h, then incubated with PM for 2 h. (**A**) phospho-p38, (**B**) phospho-ERK and (**C**) phospho-JNK. # *p* < 0.05 compared with control. * *p* < 0.05 compared with only PM exposure.

In order to understand whether PTS can inhibit PM-induced activation of MAPKs in HaCaT cells, we used Western blotting to analyze the expression of intracellular proteins. Figure 4 showed that phospho-p38 (p-p38), phospho-ERK (p-ERK) and phospho-JNK (p-JNK) expression were increased by PM treatment and significantly inhibited by 20 μM PTS.

### 3.4. PTS Inhibited PM-Induced Inflammatory, Aging and Moisturizing Proteins Expression in HaCaT Cells

**Figure 5:**
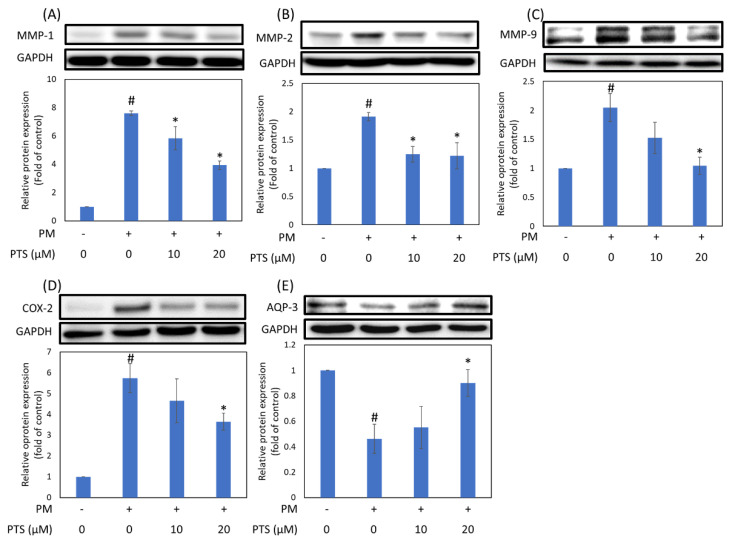
Expression of aging (MMP-1, -2 and -9), inflammatory (COX-2) and moisturizing proteins (AQP-3) following PTS treatment in PM-exposed HaCaT keratinocytes. (**A**) MMP-1, (**B**) MMP-2, (**C**) MMP-9 (**D**) COX-2 and (**E**) AQP-3. # *p* < 0.05 compared with control. * *p* < 0.05 compared with only PM exposure.

To determine whether PTS can prevent PM-induced aging and inflammation in keratinocytes, we analyzed the expression of MMPs and COX-2 proteins in HaCaT cells. As shown in Figure 5A–C, the aging marker MMP-1, MMP-2 and MMP-9 and inflammatory marker COX-2 in HaCaT cells were significantly induced by PM, while 20 μM PTS not only significantly inhibited the expression of aging proteins (MMP-1,-2 and -9), but also decreased the expression of inflammatory protein (COX-2). In the epidermis, aquaporin 3 (AQP-3) plays an important role in hydration to moisturize and enhance barrier activity [25]. Figure 5C showed that the expression of AQP-3 decreased significantly after keratinocytes were treated with PM, and this effect was reversed by 20 μM PTS.

### 3.5. The Distribution of Pterostilbene in the Intracellular and Extracellular Compartments

**Figure 6:**
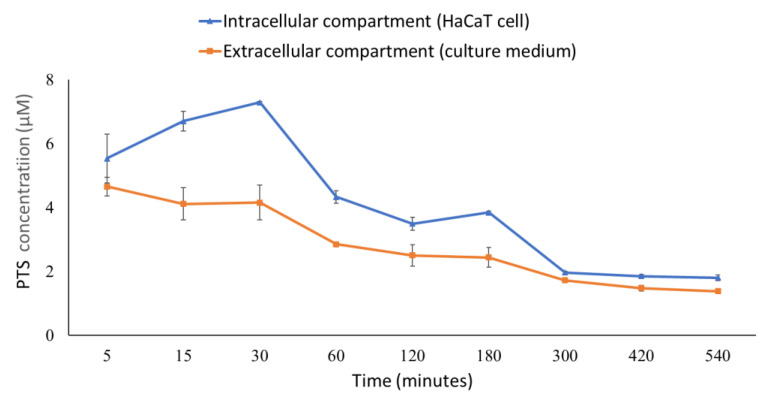
Time-dependent changes in the concentration of PTS in HaCaT cells and culture medium.

In order to confirm the content of PTS in HaCaT cells, we analyzed intracellular and extracellular PTS concentrations at different time points after treatment with PTS. The results showed that after HaCaT cells were treated with 20 μM PTS for 30 min, the intracellular PTS content reached the highest concentration of 7.29 μM (Figure 6). The intracellular PTS content decreased with time and remained at about 2 μM from 300 min to 540 min. In addition, we also collected culture medium to analyze the extracellular PTS concentration. Figure 6 showed that the extracellular PTS content decreased with time. Finally, we analyzed the PTS concentration required to inhibit various PM-induced intracellular proteins (Table 1). The nucleus protein AHR was inhibited by 4.3 to 3.5 μM intracellular PTS; the CYP1A1 and MAPKs proteins were inhibited by 3.8 to 2.0 μM intracellular PTS; the COX-2 and MMP-9 proteins were inhibited by 3.8 to 1.8 μM intracellular PTS.

### 3.6. In Vitro Skin Penetration of Pterostilbene

**Figure 7:**
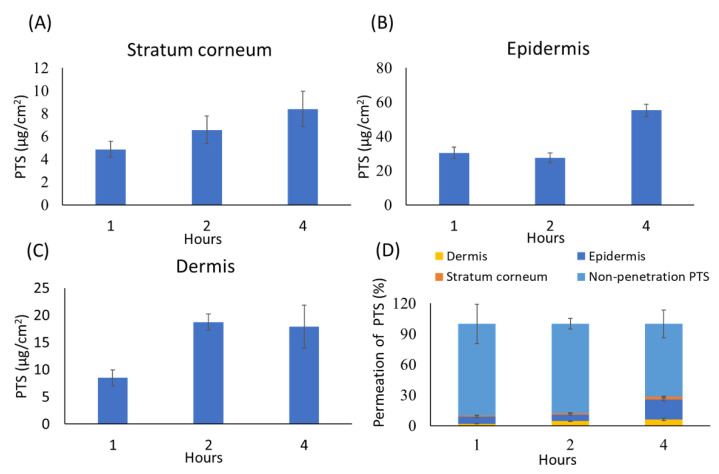
The contents of PTS in different skin layers. (**A**) Stratum corneum, (**B**) epidermis and (**C**) dermis. (**D**) The percentage of PTS that penetrated into different skin layers. Values are mean ± SD (*n* = 5).

Percutaneous absorption is the passage of active ingredients through the epidermis and dermis. Therefore, we prepared a sample essence containing 0.0625% PTS for skin absorption study and further analyzed its content in the stratum corneum, epidermis, dermis and unabsorbed compartment. As shown in Figure 7, PTS penetrated through all skin layers. In the stratum corneum (Figure 7A) and epidermis (Figure 7B), the content of PTS increased with time. In the dermis, the content of PTS was highest at two hours (Figure 7C). As shown in Figure 7D, the percent permeation of PTS reached 28.41% of the skin layer penetration at 4 h.

## 4. Discussion

Particulate matter (PM) is an important component of air pollutants, which are divided into less than 10 and 2.5 μm PM according to their diameters. Past studies have shown that PM10 can cause skin inflammation and aging by degrading collagen [9], and that PM2.5 can induce oxidative stress, subcellular organelle dysfunction and apoptosis to further damage skin cells [5]. PTS is known to have antioxidant, anti-inflammatory and anti-aging activities [26]. Recent clinical trials have shown through dermatological assessment, instrumental analysis and image analysis that PTS can reduce skin aging and brighten skin tone [27]. However, it is not known whether PTS can prevent PM-induced keratinocyte damage. The results of cell viability experiments showed that 20 and 10 μM PTS were not toxic to human keratinocyte HaCaT cells and CCD-966SK dermal fibroblast cells, respectively. In addition, 10 and 20 μM PTS significantly inhibited PM-induced intracellular ROS generation. These results suggest that PTS can effectively reduce PM-induced oxidative stress in HaCaT cells.

PAH is one of the main components of PM and can also affect human health [2]. Previous studies have indicated that PAH can induce nuclear translocation of AHR. When AHR binds to its ligand PAH, AHR translocates from the cytosol to the nucleus and forms a dimer with aryl hydrocarbon receptor nuclear translocator (ARNT) [6]. This process promotes phase I metabolism such as the transcription of cytochrome P450 family 1 subfamily A member 1 (CYP1A1), induces excessive reactive oxygen species (ROS) to cause oxidative stress, and increases production of pro-inflammatory cytokines [28,29]. Kim et al. reported that nuclear translation of AhR protein was observed after treatment with PM for 1 h [12]. Moreover, Ryu et al. reported that in HaCaT cells, AHR protein accumulated in the nucleus at 0.5 h after PM treatment [8]. Our results showed that when HaCaT cells were exposed to PM for 0.5 to 1 h, the expression of AHR protein in the nucleus was higher than at other time points (Figure 2A). At the same time, we also observed a significant decrease in AHR expression in the cytosol (Figure 2B). Furthermore, we confirmed that 20 μM PTS significantly inhibited PM-induced nuclear translocation of AHR and CYP1A1 protein expression (Figure 3).

The MAPK families have been demonstrated to regulate a variety of signaling pathways related to cell proliferation, differentiation, stress responses, apoptosis and survival. There are three main MAPK pathways in mammalian cells, namely ERK1/2, p38 and c-JUN N-terminal kinase (JNK) pathways [30,31]. Zhen et al. demonstrated that inhibition of PM-induced p-ERK, p-p38, and p-JNK protein expression in human keratinocytes could significantly inhibit PM-induced apoptosis [32]. Our results showed that pretreatment of HaCaT keratinocytes with PTS significantly inhibited the expression of PM-induced MAPKs (Figure 4). In addition, PM-induced ROS production could activate the MAPK pathway and ultimately lead to the expression of the inflammation marker protein COX-2 [12,14]. Figure 5D demonstrated that 20 μM PTS significantly inhibited COX-2 expression. Exposure of the skin to PM could lead to an increase in ROS production, which may further increase the expression of MMPs (such as MMP-1, MMP-2 and MMP-9) and induce collagen degradation to cause skin inflammation and aging [29]. Resveratrol, an analog of PTS, is known to protect against aging caused by UVB [33]. Although PTS has been shown to have anti-aging properties [34], the mechanisms of PTS in protection against PM-induced skin aging has not yet been studied. Our results indicate that PTS significantly inhibited PM-induced MMPs expression (Figure 5A–C). AQP-3 protein is abundantly expressed in the epidermis and has a moisturizing effect [35]. In addition, the intrinsic and extrinsic skin aging process and skin dryness are related to down-regulation of AQP-3 expression [36]. Tang et al. also revealed that glycolic acid can effectively attenuate UVB-induced MMP-9 overexpression and then reverse the AQP-3 expression for preventing aging and the formation of wrinkles [37]. Figure 5E demonstrated that 20 μM PTS rescued AQP-3 expression. There are many intracellular proteins in cells that have pharmacological properties. These proteins are responsible for eliminating the drug molecules and preventing toxic effects [38,39]. The key factor for drug-driven cell activity is the concentration of the compound that penetrates into the cell [40]. The current study showed that the intracellular concentration of PTS after pretreatment for 60 min is 4.3 μM, which could effectively inhibit the subsequent PM-induced nuclear translocation of AHR; PTS pretreatment for 180 min is 3.8 μM, which could effectively inhibit CYP1A1, MAPKs, COX-2 and MMPs expression (Table 1). Taken together, PTS could prevent PM-induced inflammation and aging in keratinocytes and improve moisturizing function.

A percutaneous penetration test can be used to determine the amount of chemical that has entered or been absorbed through the skin barrier, and also to assess whether there is entry into the systemic circulation [41]. Viable epidermis (VE) and the dermis of the skin are the sites where cells can metabolize drugs [42]. Most of the topical application products, such as cosmetics, can target keratinocytes, melanocytes and hair follicles in VE [43]. In this study, we used sample essence to evaluate percutaneous absorption of PTS. Figure 7 showed that PTS is mainly present in the epidermis after absorption through the skin. In addition, we did not detect PTS in the receptor fluid in the lower dermis (data not shown). These results indicate that the PTS in the essence product can penetrate the epidermis and dermis but does not enter the systemic circulation.

## 5. Conclusions

We conclude that PTS could inhibit PM-induced nuclear translocation of AHR and the expression of its main transcribed protein CYP1A1. Furthermore, PTS has the ability to improve expression of moisturizing protein and inhibit expression of inflammation and aging proteins. We observed that PTS could enter keratinocytes in the epidermal layer through percutaneous absorption and exert pharmacological activity. Therefore, PTS has the potential to become a cosmetic ingredient to prevent skin damage caused by PM exposure.

## Figures and Tables

**Table 1:** The PTS concentration required to inhibit various PM-induced intracellular proteins.

Protein	Time of PTS Administration (min)			Intracellular PTS Concentration (μM)	
	* Pre-Treat	PM Exposure	Total	PM Exposure	End of PM Exposure
AHR (nucleus)	60	30	90	4.3	between 4.3 and 3.5
CYP1A1	180	120	300	3.8	2.0
p-p38	180	120	300	3.8	2.0
p-ERK	180	120	300	3.8	2.0
p-JNK	180	120	300	3.8	2.0
COX-2	180	360	540	3.8	1.8
MMPs	180	360	540	3.8	1.8

* Pre-treat means the time of PTS treatment before exposure to PM.

## Data Availability

All data presented in the study are available on request from the corresponding author.
